# Distinct Properties of Nanofibrous Amorphous Ice

**DOI:** 10.3390/ma7127653

**Published:** 2014-11-26

**Authors:** Fanyi Cai, Chunye Xu, Jianming Zheng

**Affiliations:** Chinese Academy of Science Key Laboratory of Soft Matter Chemistry, Department of Polymer Science and Engineering, Hefei National Laboratory for Physical Sciences at Microscale, University of Science and Technology of China, Jinzhai Road 96, Hefei 230026, Anhui, China; E-Mails: hokking@mail.ustc.edu.cn (F.C.); chunye@ustc.edu.cn (C.X.)

**Keywords:** nanofibrous ice, amorphous ice, glass transition

## Abstract

We make glassy water in the form of nanofibers by electrospraying liquid water into a hyperquenching chamber. It is measured with means of differential scanning calorimetry, wide angle X-ray diffraction and Raman spectroscopy. It is found that two apparent glass transitions at *T*_g1_ = 136 K and *T*_g2_ = 228 K are detected and non-crystallized water is observed at temperatures up to 228 K. This finding may expand the research objects for liquid water at low temperatures.

## 1. Introduction

It has been a challenge for scientists to take a deep insight into the thermodynamic properties of water at a temperature between its spontaneous crystallization (*T*_x_) and homogeneous crystallization temperature (*T*_H_), where bulk water inevitably crystallizes. On one hand, bulk water can be supercooled down to *T*_H_ = 235 K without crystallization [1,2]. On the other hand, when hyperquenched glassy water is heated, it undergoes glass transition at the conventionally assigned *T*_g_ = 136 K, followed by crystallization at *T*_x_ = 150 K [3,4]. The temperature gap between *T*_x_ and *T*_H_ is so-called no man’s land of water [5]. Recent studies have found that thermodynamic response functions increase so fast that it seems to diverge at some point in this forbidden region, raising questions over whether these functions do really diverge or just experience a maximum [6,7,8,9,10]. Meanwhile, the *T*_g_ of water is also controversial. In particular, Angell and co-workers argued consistently that conventional *T*_g_ of water (136 K) should be reassigned to a higher value inside the crystalline region [11,12,13]. Furthermore, it has long been suggested that water exists in two forms: fragile and strong, and a fragile-to-strong (FS) transition [11] may fall in this forbidden region as well [14]. To avoid crystallization, recent studies have focused on water confined in nanospace where the external surface helps in restraining the water molecular motion [15,16]. The thermodynamic properties of the confined water were systematically investigated with an adiabatic calorimeter [17,18,19,20]. But it is also likely that the general structural and dynamical properties of bulk water are substantially altered in such systems [21,22].

In the present work, we make glassy water in the morphology of freestanding nanofibers by electrospraying pure water into a hyperquenching chamber. This method is reported in detail previously [23]. We also systematically exam the properties of the obtained samples over a temperature range from 120 K to 250 K with means of differential scanning calorimeter, X-ray diffraction and Raman spectroscopy.

## 2. Results and Discussion

**Figure 1:**
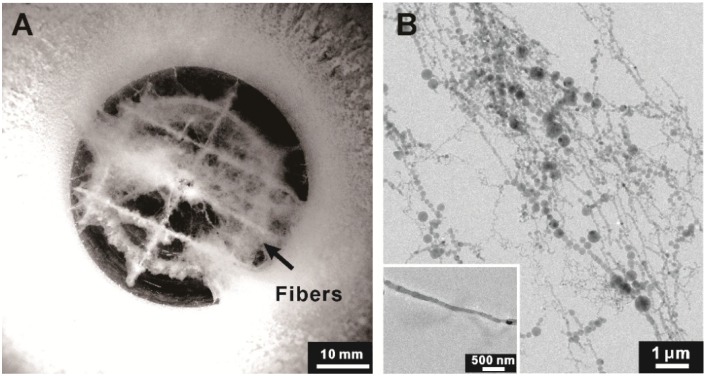
(**A**) A typical top-view image of the chamber with cotton-like ice inside suspended on a mesh near the bottom (black); (**B**) A cryo-transmission electron microscopic image of the ice. The inset presents a single ice with relatively smooth surface.

The morphology of the prepared nanofibrous ice is shown in Figure 1A, appearing in white-cotton on a mesh near the hyperquenching chamber bottom (in black). Its microscopic image is shown in Figure 1B. These ice fibers are measured to be from 40 to 400 nm in diameter. Varicose shape dominates smooth one. Occasionally, straight ice fiber with relatively smooth surface can be observed (inset in Figure 1B).

**Figure 2:**
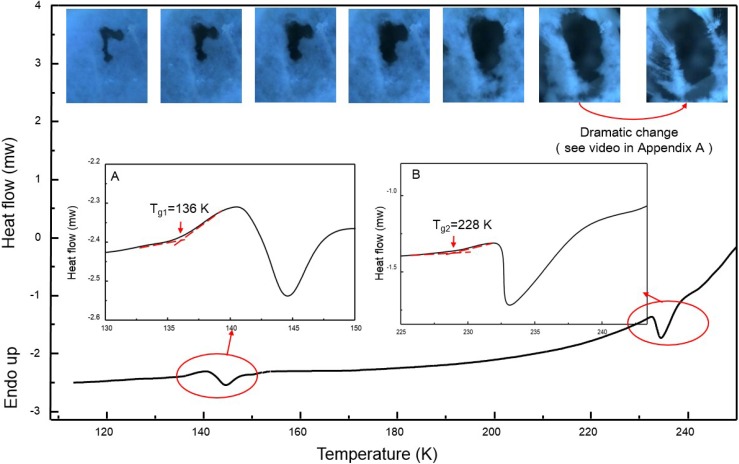
Differential scanning calorimetry (DSC) scan of the fibrous ice. The insets are the enlarged views of the heat flow around the two thermal transition temperatures at *T*_g1_ = 136 K and *T*_g2_ = 228 K. The heating rate is 30 K·min^−1^. The series of pictures present the morphological change of the intact fibrous ice in Figure 1A over spontaneously elevated temperatures. A hole throughout the ice matrix is in black.

In order to measure the thermodynamic properties of the ice, we carried out measurements with differential scanning calorimetry (DSC). The results are shown in Figure 2. Apparently, there exist two very similar thermodynamic patterns around 140 K and 230 K, showing the features of the conventional glass transition of water. By analyzing the curves as shown in the insets, the first glass transition temperature is assigned as *T*_g1_ = 136 K, which is consistent with the value reported [3,4]. The following exothermic peak immediately after *T*_g1_ centers at about 145 K, which supposedly is due to crystallization of the glassy sample to ice *I*_c_ [24]. But the area ratio of the glass transition peak to the crystallization peak is considerably large in comparison with the literature [3,24], implying that the glass transition into a more relaxed state is incomplete. Similarly, we can sign a second glass transition temperature as *T*_g2_ = 228 K. By contrast, the following exothermic peak after *T*_g2_ turns very sharp, indicating that the transition is complete. Interestingly, Maruyama *et al.* [25] have conducted an experiment on adiabatic calorimetry of water confined within nonporous of silica gel and found a C_P_ peak near 228 K at ambient pressure, and claimed it is the glass transition temperature for the internal water.

We also tried to directly observe the mechanical behaviors of the ice around the two thermal transition regions. Intact fibrous ice inside the hyper-chamber is video-recorded for its dynamic morphological change under the gravity over time at spontaneously elevated temperature. A series of the top-view images taken from the recorded video is shown in Figure 2, where the black presents a hole throughout the ice matrix. The whole heating process lasts around 2 h. As a result, both the transitions around *T*_g1_ = 136 K and *T*_g2_ = 228 K are identified. Apparently, no change was observed at all below *T*_g1_, indicating the molecular motion is frozen. However, the hole size is found to increase steadily until *T*_g2_, followed by a dramatic collapse of the nanofibrous ice (see video in Supplementary Materials). This behavior of a viscous liquid or viscoelastic body implies that any phase transition into cubic ice *I*_c_ just above *T*_g1_ is incomplete. Thus the thermal transition at *T*_g2_ = 228 K might be a liquid-liquid related transition instead of a real glass transition.

**Figure 3:**
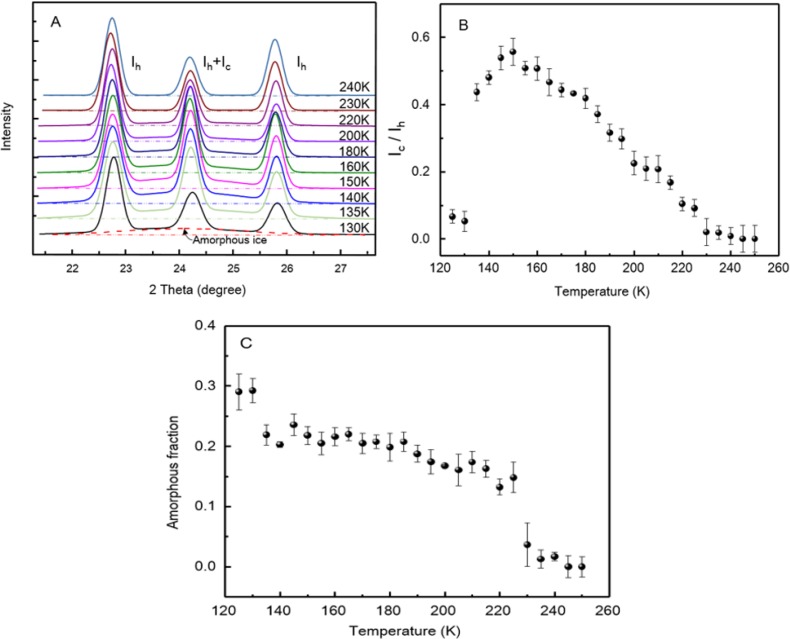
(**A**) The wide-angle X-ray diffractions (WAXD, Cu Kα) spectra changes of nanofibrous ice with increase of temperature from 130 K to 240 K; (**B**) The intensity ratio *I*_c_/*I*_h_ as a function of the temperature; (**C**) The amorphous fraction of the tested sample as a function of the temperature.

To justify our idea, we carried out measurements with wide-angle X-ray diffraction and Raman spectroscopy. The X-ray diffraction results are shown in Figure 3A. The samples were quenched with liquid nitrogen and then measured at elevated temperature up to 240 K. The Bragg peaks at about 2θ ≈ 22.7°, 24.2°, 25.8° are the (100), (002), (101) reflection of hexagonal ice *I*_h_, respectively [26], whereas the peak at 2θ ≈ 24.2° also presents the (111) reflection of cubic ice *I*_c_ [24,27]. Apparently, hexagonal form ice presents in the sample below its glass transition temperature. By analyzing the relative peak intensities, we can see the proportion change of the two different types of ice at elevated temperatures.

As shown in Figure 3B, the *I*_c_/*I*_h_ ratio presents a very small value below 130 K, but take a big jump around 140 K and then reach a peak at 150 K. Afterwards, it keeps decreasing until 230 K. The ratio change of the two form ice is the reflection of both sample purity and the *I*_c_ → *I*_h_ phase transition. On the other hand, we notice that the amorphous ice, appearing as the broad band [28], presents at all temperatures measured up to 230 K. A plot of the amorphous fraction as a function of the temperature is presented in Figure 3C. As a result, the amorphous fraction is observed to fall dramatically near *T*_g1_ and *T*_g2_, but remains relatively steady between them. This observation is distinct to these with bulk water and confined water. Actually, it is worth to point out that the tested samples are inevitably mixed with some crystallized water droplets during preparation, so the calculated amorphous fraction should be lower than the actual value of the pure fibrous ice [23].

**Figure 4:**
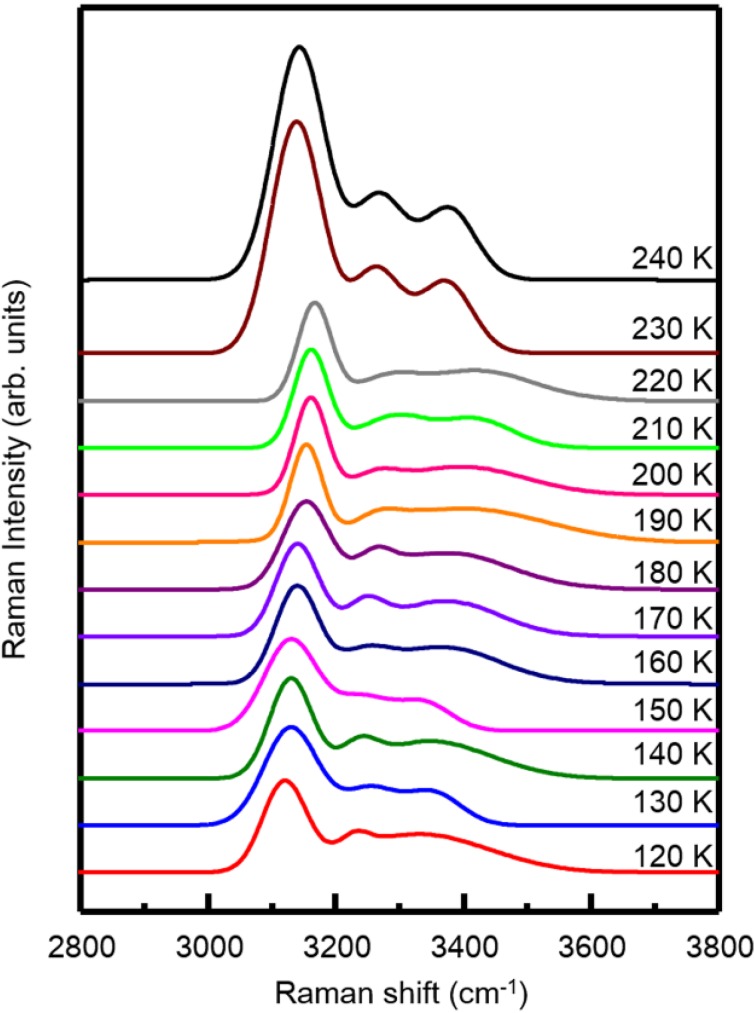
*In situ* Raman OH stretching spectral changes of the nanofibrous ice during the heating process from 120 K to 240 K.

The results measured with Raman spectroscopy are shown in Figure 4. All the obtained Raman profiles are typical ones from the coupled OH stretching vibration mode for ice [29]. According to previous reports [30], the profiles below 230 K are found to be identical to that of low density amorphous (LDA) ice; whereas these at 230 K and above presents the phase of hexagonal ice. As a result, only a slight change in peak position or intensity is observed between 130 K and 140 K, implying that the thermal transition around *T*_g1_ has a minor impact on the reconstruction of the surface water molecules of the sample. In contrast, a distinguished change in profile has been found between 220 K and 230 K, indicating that the sample has relaxed completely due to crystallization into the hexagonal form of ice *I*_h_. We also notice that the sample surfaces at least remain amorphous at all the temperatures tested, up to 220 K.

In summary, we discover the distinct properties of nanofibrous ice, which apparently embodies an incomplete glass transition at *T*_g1_ = 136 K, an amorphous/liquid state persisting throughout the conventional crystalline region and a second thermal transition at *T*_g2_ = 228 K. Interestingly, *T*_g_ = 228 K was also signed as the glass transition temperature for confined water [31]. However, the two systems seem to be quite different. Form the Raman spectra shown in Figure 4, the nanofibrous ices are actually crystal-free on their surfaces at a temperature below 220 K. Thus, our observations mostly like to be size-related phenomena of water. On one hand, the size distribution of the nanofibrous ices may result in two glass transitions due to size effects [32,33]. On the other hand, if the size effect is sufficiently enough to hinder the viscous state of water immediately after T_g1_ from crystallization [34], then the *T*_g2_ is related to the liquid-liquid transition of water [14].

## 3. Experimental Section

### 3.1. Materials

Milli-Q deionized water (resistivity: 18.2 MΩ cm) was used throughout the experiments.

### 3.2. Preparation of Nanofibrous Ice

Experimental setup is reported previously [23], which consists of an electrospray unit and a hyperquenching chamber. The former is used to produce charged micron-sized water droplets at room temperature and the latter is used to hyperquench any formed liquid threads and collect fibrous ice samples.

### 3.3. Characterization of Fibrous Ice

The morphology of the ice was characterized with a cryoEM (Tecnai F20, FEI Company, Hillsboro, OR, USA). Field emission gun of 200,000 voltage was used for electron beam generation. The images were obtained by a 4 k × 4 k multiport CCD camera (F415mp, TVIPS, Gauting, Germany) with a 4-port readout and 15 µm pixel size.

The crystal structures were monitored by *in situ* WAXD measurement at an in-house setup with a 30 W micro X-ray source (Incoatec, GmbH, Geesthacht, Germany) that provides highly parallel beam (divergence about 1 mrad) of monochromatic Cu Kα radiation (=0.154 nm). PSD-50M 1D detector was purchased from Braun Germany. Sample was placed inside an optical cryostat (THMS600, Linkam, Surrey, UK). The cryostat allows to be set between 90 and 300 K and positioned inside a working chamber purged with a dry flow of N_2_ to prevent testing sample from frosting at low temperatures.

The thermodynamic properties of the ice were measured with a DSC (DSC8000, PerkinElmer, Waltham, MA, USA). The DSC instrument was calibrated with cyclopentane and cyclohexane. DSC scans were recorded at heating rate of 30 K·min^−1^ from 113 to 298 K.

Raman spectral (LabRAM-HR, Horiba Scientific, Grenoble, France) was measured at low temperatures by a cryostat system (FDCS196, Linkam, Surrey, UK). The 514.5 nm line of argon ion laser excitation (~300 mW) was selected for use. The temperature distributions in the cooling chamber were measured by a Pt-100 thermistor (Heraeus, Hanau, Germany).

## 4. Conclusions

We have demonstrated that freestanding nanofibrous ice prepared with electrospraying unit retains in amorphous/liquid state at temperatures up to 228 K, and found two thermal transitions at *T*_g1_ = 136 K and *T*_g2_ = 228 K. We believe that the first transition at *T*_g1_ = 136 K is the glass transition water and the second one is the consequence of an incomplete phase transition near *T*_g1_ due to size effects. Our results explicitly indicate that this new approach is effective to avoid water crystallization at no costs of losing its fundamental properties.

## Supplementary Materials

A video shows the dynamic morphological change of the ice fibers under the gravity over time at spontaneously elevated temperature. The real process lasts around 2 h and the presented video is fast forward.

Supplementary materials can be accessed at: http://www.mdpi.com/1996-1944/7/12/7653/s1.
